# Supplementary material for: The LuxO-OpaR quorum-sensing cascade differentially controls Vibriophage VP882 lysis-lysogeny decision making in liquid and on surfaces
Source: PLoS Genet. 2024 Jul 30;20(7):e1011243. doi: 10.1371/journal.pgen.1011243 (PMC11315295; doi:10.1371/journal.pgen.1011243)
Supplement: S1 Table — (DOCX) [file pgen.1011243.s001.docx]

**S1 Table. Strains used in this study.**

| **Strain** | **Identifier** | **Genotype** | **Reference** |
| --- | --- | --- | --- |
| *V. parahaemolyticus* O3 K6 RIMD 2210633 (RIMD) | FJS-S0045 | Wildtype O3 K6 clinical isolate (WT, Sm^r^) | (1) |
|  | FJS-S0133 | *luxO^D61E^* | This study |
|  | FJS-S0150 | *luxO^D61A^* | This study |
|  | FJS-S0176 | Δ*opaR* | This study |
|  | FJS-S0178 | *luxO^D61E^* Δ*opaR* | This study |
|  | FJS-S0445 | *luxO^D61A^* Δ*opaR* | This study |
|  | FJS-S0277 | Δ*cpsA* | This study |
|  | FJS-S0279 | Δ*cpsA luxO^D61E^* | This study |
|  | FJS-S0285 | Δ*cpsA luxO^D61A^* | This study |
|  | FJS-S0281 | Δ*cpsA* Δ*opaR* | This study |
|  | FJS-S0283 | Δ*cpsA luxO^D61E^* Δ*opaR* | This study |
|  | FJS-S0427 | Δ*cpsA luxO^D61A^* Δ*opaR* | This study |
|  | FJS-S0289 | Δ*cpsA* Δ*pomA* | This study |
|  | FJS-S0291 | Δ*cpsA* Δ*pomA luxO^D61E^* | This study |
|  | FJS-S0297 | Δ*cpsA* Δ*pomA luxO^D61A^* | This study |
|  | FJS-S0293 | Δ*cpsA* Δ*pomA* Δ*opaR* | This study |
|  | FJS-S0295 | Δ*cpsA* Δ*pomA luxO^D61E^* Δ*opaR* | This study |
|  | FJS-S0428 | Δ*cpsA* Δ*pomA luxO^D61A^* Δ*opaR* | This study |
|  | FJS-S0946 | Δ*cpsA*/pEVS143-P*_luxC_*-*luxCDABE* | This study |
|  | FJS-S0947 | Δ*cpsA luxO^D61E^*/pEVS143-P*_luxC_*-*luxCDABE* | This study |
|  | FJS-S0950 | Δ*cpsA luxO^D61A^*/pEVS143-P*_luxC_*-*luxCDABE* | This study |
|  | FJS-S0948 | Δ*cpsA* Δ*opaR*/pEVS143-P*_luxC_*-*luxCDABE* | This study |
|  | FJS-S0949 | Δ*cpsA luxO^D61E^* Δ*opaR*/pEVS143-P*_luxC_*-*luxCDABE* | This study |
|  | FJS-S0951 | Δ*cpsA luxO^D61A^* Δ*opaR*/pEVS143-P*_luxC_*-*luxCDABE* | This study |
|  | FJS-S0553 | Δ*cpsA* Δ*pomA*/pEVS143-P*_bad_*-*q* | This study |
|  | FJS-S0554 | Δ*cpsA* Δ*pomA luxO^D61E^*/pEVS143-P*_bad_*-*q* | This study |
|  | FJS-S0557 | Δ*cpsA* Δ*pomA luxO^D61A^*/pEVS143-P*_bad_*-*q* | This study |
|  | FJS-S0555 | Δ*cpsA* Δ*pomA* Δ*opaR*/pEVS143-P*_bad_*-*q* | This study |
|  | FJS-S0556 | Δ*cpsA* Δ*pomA luxO^D61E^* Δ*opaR*/pEVS143-P*_bad_*-*q* | This study |
|  | FJS-S0558 | Δ*cpsA* Δ*pomA luxO^D61A^* Δ*opaR*/pEVS143-P*_bad_*-*q* | This study |
|  | FJS-S1033 | Δ*cpsA* Δ*pomA*(φVP882::*Cm^r^*)/pEVS143-P*_bad_*-*q* | This study |
|  | FJS-S1034 | Δ*cpsA* Δ*pomA luxO^D61E^*(φVP882::*Cm^r^*)/pEVS143-P*_bad_*-*q* | This study |
|  | FJS-S1037 | Δ*cpsA* Δ*pomA luxO^D61A^*(φVP882::*Cm^r^*)/pEVS143-P*_bad_*-*q* | This study |
|  | FJS-S1035 | Δ*cpsA* Δ*pomA* Δ*opaR*(φVP882::*Cm^r^*)/pEVS143-P*_bad_*-*q* | This study |
|  | FJS-S1036 | Δ*cpsA* Δ*pomA luxO^D61E^* Δ*opaR*(φVP882::*Cm^r^*)/pEVS143-P*_bad_*-*q* | This study |
|  | FJS-S1038 | Δ*cpsA* Δ*pomA luxO^D61A^* Δ*opaR*(φVP882::*Cm^r^*)/pEVS143-P*_bad_*-*q* | This study |
|  | FJS-S1151 | Δ*cpsA* Δ*pomA*(φVP882)/pEVS143-P*_bad_*-*q* | This study |
|  | FJS-S1153 | Δ*cpsA* Δ*pomA luxO^D61E^*(φVP882)/pEVS143-P*_bad_*-*q* | This study |
|  | FJS-S1157 | Δ*cpsA* Δ*pomA luxO^D61A^*(φVP882)/pEVS143-P*_bad_*-*q* | This study |
|  | FJS-S1155 | Δ*cpsA* Δ*pomA* Δ*opaR*(φVP882)/pEVS143-P*_bad_*-*q* | This study |
|  | FJS-S0843 | Δ*cpsA* Δ*pomA*/pEVS143-P*_bad_*-*q*/pXBCm | This study |
|  | FJS-S0845 | Δ*cpsA* Δ*pomA*/pEVS143-P*_bad_*-*q*/pXBCm-*qrr*2 | This study |
|  | FJS-S0849 | Δ*cpsA* Δ*pomA luxO^D61E^*/pEVS143-P*_bad_*-*q*/pXBCm | This study |
|  | FJS-S0851 | Δ*cpsA* Δ*pomA luxO^D61E^*/pEVS143-P*_bad_*-*q*/pXBCm-*qrr*2 | This study |
|  | FJS-S0855 | Δ*cpsA* Δ*pomA luxO^D61A^*/pEVS143-P*_bad_*-*q*/pXBCm | This study |
|  | FJS-S0857 | Δ*cpsA* Δ*pomA luxO^D61A^*/pEVS143-P*_bad_*-*q*/pXBCm-*qrr*2 | This study |
|  | FJS-S1059 | Δ*cpsA* Δ*pomA*/pEVS143-P*_qrr_*_1_-*luxCDABE* | This study |
|  | FJS-S1065 | Δ*cpsA* Δ*pomA*/pEVS143-P*_qrr_*_2_-*luxCDABE* | This study |
|  | FJS-S1071 | Δ*cpsA* Δ*pomA*/pEVS143-P*_qrr_*_3_-*luxCDABE* | This study |
|  | FJS-S1077 | Δ*cpsA* Δ*pomA*/pEVS143-P*_qrr_*_4_-*luxCDABE* | This study |
|  | FJS-S1083 | Δ*cpsA* Δ*pomA*/pEVS143-P*_qrr_*_5_-*luxCDABE* | This study |
|  | FJS-S1061 | Δ*cpsA* Δ*pomA luxO^D61A^*/pEVS143-P*_qrr_*_1_-*luxCDABE* | This study |
|  | FJS-S1067 | Δ*cpsA* Δ*pomA luxO^D61A^*/pEVS143-P*_qrr_*_2_-*luxCDABE* | This study |
|  | FJS-S1073 | Δ*cpsA* Δ*pomA luxO^D61A^*/pEVS143-P*_qrr_*_3_-*luxCDABE* | This study |
|  | FJS-S1079 | Δ*cpsA* Δ*pomA luxO^D61A^*/pEVS143-P*_qrr_*_4_-*luxCDABE* | This study |
|  | FJS-S1085 | Δ*cpsA* Δ*pomA luxO^D61A^*/pEVS143-P*_qrr_*_5_-*luxCDABE* | This study |
|  | FJS-S0988 | Δ*cpsA*/pEVS143-P*_luxC_*-*luxCDABE*/pXBCm | This study |
|  | FJS-S0990 | Δ*cpsA*/pEVS143-P*_luxC_*-*luxCDABE*/pXBCm-*qrr*2 | This study |
|  | FJS-S0565 | Δ*cpsA* Δ*pomA* Δ*scrABC*/pEVS143-P*_bad_*-*q* | This study |
|  | FJS-S0566 | Δ*cpsA* Δ*pomA* Δ*scrABC luxO^D61E^*/pEVS143-P*_bad_*-*q* | This study |
|  | FJS-S0569 | Δ*cpsA* Δ*pomA* Δ*scrABC luxO^D61A^*/pEVS143-P*_bad_*-*q* | This study |
|  | FJS-S0567 | Δ*cpsA* Δ*pomA* Δ*scrABC* Δ*opaR*/pEVS143-P*_bad_*-*q* | This study |
|  | FJS-S0568 | Δ*cpsA* Δ*pomA* Δ*scrABC luxO^D61E^* Δ*opaR*/pEVS143-P*_bad_*-*q* | This study |
|  | FJS-S0570 | Δ*cpsA* Δ*pomA* Δ*scrABC luxO^D61A^* Δ*opaR*/pEVS143-P*_bad_*-*q* | This study |
|  | FJS-S952 | Δ*cpsA* Δ*pomA*/pEVS143-P*_bad_*-*q*/pXBCm-*opaR* | This study |
|  | FJS-S0883 | Δ*cpsA* Δ*pomA* Δ*opaR*/pEVS143-P*_bad_*-*q*/pXBCm | This study |
|  | FJS-S0954 | Δ*cpsA* Δ*pomA* Δ*opaR*/pEVS143-P*_bad_*-*q*/pXBCm-*opaR* | This study |
|  | FJS-S0994 | Δ*cpsA*/pEVS143-P*_luxC_*-*luxCDABE*/pXBCm-*opaR* | This study |
|  | FJS-S0995 | Δ*cpsA* Δ*opaR*/pEVS143-P*_luxC_*-*luxCDABE*/pXBCm | This study |
|  | FJS-S0996 | Δ*cpsA* Δ*opaR*/pEVS143-P*_luxC_*-*luxCDABE*/pXBCm-*opaR* | This study |
|  | FJS-S0933 | Δ*pomA* Δ*ebgRA*::P*_tac_*-*mScarletI* /pEVS143-P*_cpsA_*-*luxCDABE*/pXBCm | This study |
|  | FJS-S0934 | Δ*pomA* Δ*ebgRA*::P*_tac_*-*mScarletI* /pEVS143-P*_cpsA_*-*luxCDABE*/pXBCm-*opaR* | This study |
|  | FJS-S0935 | Δ*pomA* Δ*opaR* Δ*ebgRA*::P*_tac_*-*mScarletI* /pEVS143-P*_cpsA_*-*luxCDABE*/pXBCm | This study |
|  | FJS-S0936 | Δ*pomA* Δ*opaR* Δ*ebgRA*::P*_tac_*-*mScarletI* /pEVS143-P*_cpsA_*-*luxCDABE*/pXBCm-*opaR* | This study |
|  | FJS-S0958 | Δ*cpsA* Δ*pomA* Δ*scrABC*/pEVS143-P*_bad_*-*q*/pXBCm | This study |
|  | FJS-S0964 | Δ*cpsA* Δ*pomA* Δ*scrABC*/pEVS143-P*_bad_*-*q*/pXBCm-*scrABC* | This study |
|  | FJS-S1031 | Δ*cpsA* Δ*pomA* Δ*opaR*/pEVS143-P*_bad_*-*q*/pXBCm-*scrC^E554A^* | This study |
|  | FJS-S1094 | Δ*cpsA* Δ*pomA* Δ*scrABC*/pEVS143-P*_bad_*-*q*/pXBCm-*tpdA* | This study |
|  | FJS-S1095 | Δ*cpsA* Δ*pomA* Δ*opaR*/pEVS143-P*_bad_*-*q*/pXBCm-*gefA* | This study |
|  | FJS-S0820 | Δ*pomA* Δ*ebgRA*::P*_tac_*-*mScarletI* /pEVS143-P*_scrA_*-*luxCDABE* | This study |
|  | FJS-S0821 | Δ*pomA luxO^D61E^* Δ*ebgRA*::P*_tac_*-*mScarletI* /pEVS143-P*_scrA_*-*luxCDABE* | This study |
|  | FJS-S0824 | Δ*pomA luxO^D61A^* Δ*ebgRA*::P*_tac_*-*mScarletI* /pEVS143-P*_scrA_*-*luxCDABE* | This study |
|  | FJS-S0822 | Δ*pomA* Δ*opaR* Δ*ebgRA*::P*_tac_*-*mScarletI* /pEVS143-P*_scrA_*-*luxCDABE* | This study |
|  | FJS-S0823 | Δ*pomA luxO^D61E^* Δ*opaR* Δ*ebgRA*::P*_tac_*-*mScarletI* /pEVS143-P*_scrA_*-*luxCDABE* | This study |
|  | FJS-S0825 | Δ*pomA luxO^D61A^* Δ*opaR* Δ*ebgRA*::P*_tac_*-*mScarletI* /pEVS143-P*_scrA_*-*luxCDABE* | This study |
|  | FJS-S0360 | Δ*pomA*/pFY4535 | This study |
|  | FJS-S0362 | Δ*pomA luxO^D61E^*/pFY4535 | This study |
|  | FJS-S0364 | Δ*pomA* Δ*opaR*/pFY4535 | This study |
|  | FJS-S0366 | Δ*pomA luxO^D61E^* Δ*opaR*/pFY4535 | This study |
|  | FJS-S1053 | Δ*pomA* Δ*opaR*/pFY4535/pXBCm | This study |
|  | FJS-S1054 | Δ*pomA* Δ*opaR*/pFY4535/pXBCm-*scrABC* | This study |
|  | FJS-S1055 | Δ*pomA* Δ*opaR*/pFY4535/pXBCm-*scrC^E554A^* | This study |
|  | FJS-S1020 | Δ*pomA* Δ*scrABC* Δ*ebgRA*::P*_tac_*-*mScarletI*/pEVS143-P*_lafA_*-*luxCDABE*/pXBCm | This study |
|  | FJS-S1024 | Δ*pomA* Δ*scrABC* Δ*ebgRA*::P*_tac_*-*mScarletI*/pEVS143-P*_lafA_*-*luxCDABE*/pXBCm-*scrABC* | This study |
|  | FJS-S1019 | Δ*pomA* Δ*opaR* Δ*ebgRA*::P*_tac_*-*mScarletI*/pEVS143-P*_lafA_*-*luxCDABE*/pXBCm | This study |
|  | FJS-S1029 | Δ*pomA* Δ*opaR* Δ*ebgRA*::P*_tac_*-*mScarletI*/pEVS143-P*_lafA_*-*luxCDABE*/pXBCm-*scrC^E554A^* | This study |
|  | FJS-S1098 | Δ*pomA* Δ*scrABC* Δ*ebgRA*::P*_tac_*-*mScarletI*/pEVS143-P*_lafA_*-*luxCDABE*/pXBCm-*tpdA* | This study |
|  | FJS-S1101 | Δ*pomA* Δ*opaR* Δ*ebgRA*::P*_tac_*-*mScarletI*/pEVS143-P*_lafA_*-*luxCDABE*/pXBCm-*gefA* | This study |
| *V. parahaemolyticus* O3 K6 882 | FJS-S0018 | Wildtype φVP882 lysogen | (2) |
|  | BB-Vp0004 | (φVP882)/pEVS143-P*_bad_-vqmAφ* | (3) |
| *E. coli* TOP10 | FJS-S0970 | /pEVS143-P*_bad_*-*opaR*-*5’UTR*-*gfp*/pXBCm | This study |
|  | FJS-S0972 | /pEVS143-P*_bad_*-*opaR*-*5’UTR*-*gfp*/pXBCm-*qrr*2 | This study |

**REFERENCES**

1. Makino K, Oshima K, Kurokawa K, Yokoyama K, Uda T, Tagomori K, et al. Genome sequence of *Vibrio parahaemolyticus*: a pathogenic mechanism distinct from that of *V cholerae*. The Lancet. 2003;361(9359):743–9.

2. Lan SF, Huang CH, Chang CH, Liao WC, Lin IH, Jian WN, et al. Characterization of a New Plasmid-Like Prophage in a Pandemic *Vibrio parahaemolyticus* O3:K6 Strain. Appl Environ Microbiol. 2009;75(9):2659–67.

3. Silpe JE, Bassler BL. A Host-Produced Quorum-Sensing Autoinducer Controls a Phage Lysis-Lysogeny Decision. Cell. 2019;176(1–2):268-280.e13.
