# Supplementary material for: The LuxO-OpaR quorum-sensing cascade differentially controls Vibriophage VP882 lysis-lysogeny decision making in liquid and on surfaces
Source: PLoS Genet. 2024 Jul 30;20(7):e1011243. doi: 10.1371/journal.pgen.1011243 (PMC11315295; doi:10.1371/journal.pgen.1011243)
Supplement: S2 Table — (DOCX) [file pgen.1011243.s002.docx]

**S2 Table. Primers and Synthesized Fragments used in this study.**

| **Primer*** | **Sequence**** | | | **Description/Associated construct** |
| --- | --- | --- | --- | --- |
| FJS-O132 | | agtgaactgcatgaattcccGTGGAAGCAATGACCATG | Forward primer for upstream homology arm for the *cpsA* deletion / pRE112-Δ*cpsA* | |
| FJS-O133 | | cgccgccttaCATGACCTAGTTTCCCTTC | Reverse primer for upstream homology arm for the *cpsA* deletion / pRE112-Δ*cpsA* | |
| FJS-O134 | | ctaggtcatgTAAGGCGGCGATGATGAAAAC | Forward primer for downstream homology arm for the *cpsA* deletion / pRE112-Δ*cpsA* | |
| FJS-O135 | | atgcgatatcgagctctcccGCTGTTCCAATCGTGTTTG | Reverse primer for downstream homology arm for the *cpsA* deletion / pRE112-Δ*cpsA* | |
| FJS-O138 | | GCCGTACACTAGTATCACAACC | Forward primer for verification of the *cpsA* deletion | |
| FJS-O139 | | CACGACTAAACGATGACGATGA | Reverse primer for verification of the *cpsA* deletion | |
| FJS-O018 | | agtgaactgcatgaattcccATGCTTGGCCGACACACATG | Forward primer for upstream homology arm for the *pomA* deletion / pRE112-Δ*pomA* | |
| FJS-O019 | | tccgcgattaCACAAAGCACTCCTCACGC | Reverse primer for upstream homology arm for the *pomA* deletion / pRE112-Δ*pomA* | |
| FJS-O020 | | gtgctttgtgTAATCGCGGAGATTTGTG | Forward primer for downstream homology arm for the *pomA* deletion / pRE112-Δ*pomA* | |
| FJS-O021 | | atgcgatatcgagctctcccTTTATCTTCTGAACTATTTTATAGACG | Reverse primer for downstream homology arm for the *pomA* deletion / pRE112-Δ*pomA* | |
| FJS-O022 | | ATAGCGTGAGGAGTGCTTTG | Forward primer for verification of the *pomA* deletion | |
| FJS-O023 | | GGCGTGTGAGTCAGGATTT | Reverse primer for verification of the *pomA* deletion | |
| FJS-O036 | | agtgaactgcatgaattcccCTTAGTGGGTATCAACTTGC | Forward primer for upstream homology arm for the *luxO* point mutants / pRE112-*luxO^D61x^* | |
| FJS-O037 | | gacgaagctcGAGAAGAATAAGATCTGGAATTCG | Reverse primer for upstream homology arm for the *luxO^D61E^* point mutant / pRE112-*luxO^D61E^* | |
| FJS-O040 | | gacgaagtgcGAGAAGAATAAGATCTGGAATTCG | Reverse primer for upstream homology arm for the *luxO^D61A^* point mutant / pRE112-*luxO^D61A^* | |
| FJS-O038 | | tattcttctcgagCTTCGTCTGCCAGATATGAC | Forward primer for downstream homology arm for the *luxO^D61E^* point mutant / pRE112-*luxO^D61E^* | |
| FJS-O041 | | tattcttctcgcaCTTCGTCTGCCAGATATGAC | Forward primer for downstream homology arm for the *luxO^D61A^* point mutant / pRE112-*luxO^D61A^* | |
| FJS-O039 | | atgcgatatcgagctctcccGCTCAATCAGTTTAGATACAGATG | Reverse primer for downstream homology arm for the *luxO* point mutants / pRE112-*luxO^D61x^* | |
| FJS-O067 | | GCTTTTTAGCGCATGGCTGATCTC | Forward primer for verification of the *luxO* point mutants | |
| FJS-O068 | | GAGGGGTCGCTAATATATCAGCATGC | Reverse primer for verification of the *luxO* point mutants | |
| FJS-O046 | | agtgaactgcatgaattcccAGACCGTTGAAGCATCGTAC | Forward primer for upstream homology arm for the *opaR* deletion / pRE112-Δ*opaR* | |
| FJS-O047 | | ctgagctttaCATATCCATTTTCCTTGCCATTTG | Reverse primer for upstream homology arm for the *opaR* deletion / pRE112-Δ*opaR* | |
| FJS-O048 | | aatggatatgTAAAGCTCAGATTTGAACACG | Forward primer for downstream homology arm for the *opaR* deletion / pRE112-Δ*opaR* | |
| FJS-O049 | | atgcgatatcgagctctcccGGTCTAGAAATGGGTACGG | Reverse primer for downstream homology arm for the *opaR* deletion / pRE112-Δ*opaR* | |
| FJS-O050 | | GATACCAACACCAACAACGAAC | Forward primer for verification of the *opaR* deletion | |
| FJS-O051 | | CAATCACTGACCTGCCAAATAAA | Reverse primer for verification of the *opaR* deletion | |
| FJS-O199 | | gcggtgtaagtgaactgcatgaattcccTGATTATCACCGCCAGCATG | Forward primer for upstream homology arm for the *scrABC* deletion / pRE112-Δ*scrABC* | |
| FJS-O200 | | attgaaagaagttaCATTTTTTTCGATCCTTGTCGG | Reverse primer for upstream homology arm for the *scrABC* deletion / pRE112-Δ*scrABC* | |
| FJS-O201 | | gatcgaaaaaaatgTAACTTCTTTCAATACAACCTC | Forward primer for downstream homology arm for the *scrABC* deletion / pRE112-Δ*scrABC* | |
| FJS-O202 | | ggtaccgcatgcgatatcgagctctcccTCAATCACTTCCGCTTTAC | Reverse primer for downstream homology arm for the *scrABC* deletion / pRE112-Δ*scrABC* | |
| FJS-O234 | | GACCGTGAGTTGCGATGTAA | Forward primer for verification of the *scrABC* deletion | |
| FJS-O235 | | GGCTTGCTGTTGAGAGGTAA | Reverse primer for verification of the *scrABC* deletion | |
| FJS-O329 | | AATTCGATATCAAGCTTATCGATAC | Forward primer for generation of linearized pXBCm | |
| FJS-O330 | | AGCTCCCATTTCACTTTTC | Reverse primer for generation of linearized pXBCm | |
| FJS-O337 | | tcagtgatagagaaaagtgaaatgggagctCGACCCTTCTTAAGCCGAG | Forward primer for insertion of the *qrr*2 gene into pXBCm / pXBCm-*qrr*2 | |
| FJS-O338 | | cgacggtatcgataagcttgatatcgaattTAAATCAAATAACCTTAGTAAAGAAATGG | Reverse primer for insertion of the *qrr*2 gene into pXBCm / pXBCm-*qrr*2 | |
| FJS-O387 | | tcagtgatagagaaaagtgaaatgggagct**gtacaggaggtgtgaa**ATGGACTCAATTGCAAAGAG | Forward primer for insertion of the *opaR* gene into pXBCm / pXBCm-*opaR* | |
| FJS-O388 | | cgacggtatcgataagcttgatatcgaattTTAGTGTTCGCGATTGTAGATG | Reverse primer for insertion of the *opaR* gene into pXBCm / pXBCm-*opaR* | |
| FJS-O345 | | tcagtgatagagaaaagtgaaatgggagct**gtacaggaggtgtgaa**ATGAGCGACAAGGATTCTATTC | Forward primer for insertion of the *scrABC* genes into pXBCm / pXBCm-*scrABC* | |
| FJS-O346 | | cgacggtatcgataagcttgatatcgaattTTATGACCAAGTAGGTTGGTTTAG | Reverse primer for insertion of the *scrABC* genes into pXBCm / pXBCm-*scrABC* | |
| FJS-O389 | | tcagtgatagagaaaagtgaaatgggagct**gtacaggaggtgtgaa**ATGAAAAAGGCTGTTAAAAAAATATC | Forward primer for insertion of the *scrC* gene into pXBCm / pXBCm-*scrC^E554A^* | |
| FJS-O410 | | ccaacgcatcaatgcTGCTGCGCCAACAATTTTG | Reverse primer to generate *scrC^E554A^* / pXBCm-*scrC^E554A^* | |
| FJS-O411 | | attgttggcgcagcaGCATTGATGCGTTGGAATG | Forward primer to generate *scrC^E554A^* / pXBCm-*scrC^E554A^* | |
| FJS-O390 | | cgacggtatcgataagcttgatatcgaattTTATGACCAAGTAGGTTGGTTTAG | Reverse primer for insertion of the *scrC* gene into pXBCm / pXBCm-*scrC^E554A^* | |
| FJS-O442 | | agaaaagtgaaatgggagct**gtacaggaggtgtgaa**ATGATTAGGTTTGAACTTGGAAAC | Forward primer for insertion of the *tpdA* gene into pXBCm / pXBCm-*tpdA* | |
| FJS-O443 | | cgacggtatcgataagcttgatatcgaattCTAGAAATGCAGAGGATAGAAG | Reverse primer for insertion of the *tpdA* gene into pXBCm / pXBCm-*tpdA* | |
| FJS-O440 | | gagaaaagtgaaatgggagct**gtacaggaggtgtgaa**ATGACTGACGAGTTTAAGAAATC | Forward primer for insertion of the *gefA* gene into pXBCm / pXBCm-*gefA* | |
| FJS-O441 | | cgacggtatcgataagcttgatatcgaattTTAGATAGGCATCACTCGG | Reverse primer for insertion of the *gefA* gene into pXBCm / pXBCm-*gefA* | |
| FJS-O062 | | ATGACTAAAAAAATTTCATTCATTATTAAC | Forward primer for generation of linearized pEVS143-*luxCDABE* | |
| FJS-O025 | | TTAATTAACTCGAGCGGTACC | Reverse primer for generation of linearized pEVS143-*luxCDABE* | |
| FJS-O407 | | catgcggcgggtaccgctcgagttaattaaGTGGTTTCTTATGAAGTCCATAC | Forward primer for the insertion of *luxC* promoter from *V. campbellii* into pEVS143-*luxCDABE* (1) / pEVS143-P*_luxC_*-*luxCDABE* | |
| FJS-O408 | | gttaataatgaatgaaatttttttagtcatttcTTGCCCATTTATTATTAAAGGTAAG | Reverse primer for insertion of the *luxC* promoter from *V. campbellii* into pEVS143-*luxCDABE* (1) / pEVS143-P*_luxC_*-*luxCDABE* | |
| FJS-O428 | | catgcggcgggtaccgctcgagttaattaaTAACGGCGTGAGGTACGAAC | Forward primer for insertion of the *qrr*1 promoter into pEVS143-*luxCDABE* / pEVS143-P*_qrr_*_1_-*luxCDABE* | |
| FJS-O429 | | gttaataatgaatgaaatttttttagtcat**ttcacacctcctgtac**CTAATATATCAGCATGCTTTATGCC | Reverse primer for insertion of the *qrr*1 promoter into pEVS143-*luxCDABE* / pEVS143-P*_qrr_*_1_-*luxCDABE* | |
| FJS-O430 | | catgcggcgggtaccgctcgagttaattaaAGTGGTTGCTTATGAATCAATC | Forward primer for insertion of the *qrr*2 promoter into pEVS143-*luxCDABE* / pEVS143-P*_qrr_*_2_-*luxCDABE* | |
| FJS-O431 | | gttaataatgaatgaaatttttttagtcat**ttcacacctcctgtac**AGAAGTATTATGCATTAATCATGCC | Reverse primer for insertion of the *qrr*2 promoter into pEVS143-*luxCDABE* / pEVS143-P*_qrr_*_2_-*luxCDABE* | |
| FJS-O432 | | catgcggcgggtaccgctcgagttaattaaCAGCCTTAGCAGGCTCGG | Forward primer for insertion of the *qrr*3 promoter into pEVS143-*luxCDABE* / pEVS143-P*_qrr_*_3_-*luxCDABE* | |
| FJS-O433 | | gttaataatgaatgaaatttttttagtcat**ttcacacctcctgtac**ATTTATATAATGCAGTTACTGTGCCAAC | Reverse primer for insertion of the *qrr*3 promoter into pEVS143-*luxCDABE* / pEVS143-P*_qrr_*_3_-*luxCDABE* | |
| RIMD *qrr*4 promoter | | ggcgggtaccgctcgagttaattaaCTGAGGTATTTTTCCTCATTAATTAGCAGTATAGGGTAAAAATATGAAAGCTAATGGATAATCATAAAGTAGTAGTTGGTTTTTTGCTGAGAAAGTGATTAGTAGCAATGTAACAAGTGGCATATTTGCATGCTATTGCATTTTGCAAATGCAATTTGCGAAAGTGCTGGTTAATAATGCGTCGATATGCACCTGGATCTATTAAAAAACGGCTTTTTTAAAGTTGGCACGCATCGTGCTTTATCTAGAGGTACAGGAGGTGTGAA**gtacaggaggtgtgaa**atgactaaaaaaatttcattcatta | Synthetic fragment for insertion of the *qrr*4 promoter region into pEVS143-*luxCDABE* / pEVS143-P*_qrr_*_4_-*luxCDABE* | |
| FJS-O436 | | catgcggcgggtaccgctcgagttaattaaGCAGCTGACGTTTCTCGTG | Forward primer for insertion of the *qrr*5 promoter into pEVS143-*luxCDABE* / pEVS143-P*_qrr_*_5_-*luxCDABE* | |
| FJS-O437 | | gttaataatgaatgaaatttttttagtcat**ttcacacctcctgtac**ATAGTACTAAAGCATGAGGCG | Reverse primer for insertion of the *qrr*5 promoter into pEVS143-*luxCDABE* / pEVS143-P*_qrr_*_5_-*luxCDABE* | |
| FJS-O359 | | catgcggcgggtaccgctcgagttaattaaATTATTCACCTATAGTTGTTATAAATCC | Forward primer for insertion of the *cpsA* promoter into pEVS143-*luxCDABE* / pEVS143-P*_cpsA_*-*luxCDABE* | |
| FJS-O360 | | gttaataatgaatgaaatttttttagtcatGACCTAGTTTCCCTTCTAGC | Reverse primer for insertion of the *cpsA* promoter into pEVS143-*luxCDABE* / pEVS143-P*_cpsA_*-*luxCDABE* | |
| FJS-O250 | | catgcggcgggtaccgctcgagttaattaaAGCCATTTTATGAAACTTAACATATTG | Forward primer for insertion of the *scrA* promoter into pEVS143-*luxCDABE* / pEVS143-P*_scrA_*-*luxCDABE* | |
| FJS-O251 | | gttaataatgaatgaaatttttttagtcatTTTTTTCGATCCTTGTCGG | Reverse primer for the insertion of the *scrA* promoter into pEVS143-*luxCDABE* / pEVS143-P*_scrA_*-*luxCDABE* | |
| FJS-O063 | | gtaccgctcgagttaattaaTACTTTTCTCGTTTTAGATTTTC | Forward primer for insertion of the *lafA* promoter into pEVS143-*luxCDABE* / pEVS143-P*_lafA_*-*luxCDABE* | |
| FJS-O064 | | gaatgaaatttttttagtcatCTTAGTCTCCTTAGTTTATCAC | Reverse primer for insertion of the *lafA* promoter into pEVS143-*luxCDABE* / pEVS143-P*_lafA_*-*luxCDABE* | |
| FJS-S371 | | CGCAACCAGGATCCGGTG | Forward primer for generation of linearized pEVS143-*araC-*P*_bad_* | |
| FJS-S372 | | ATGGAGAAACAGTAGAGAGTTGC | Reverse primer for generation of linearized pEVS143-*araC-*P*_bad_* | |
| FJS-S375 | | ttttatcgcaactctctactgtttctccatTGCTTAAAGCAATTATTAAAATAATCAATTAG | Forward primer for insertion of the *opaR* 5’UTR region into pEVS143-*araC-*P*_bad_* / pEVS143-*araC-*P*_bad_*-*opaR*-*5’UTR*-*gfp* | |
| FJS-S376 | | tttagacatggtaccAGTTCTAGGTCTCTTTGCAATTG | Reverse primer for insertion of the *opaR* 5’UTR region into pEVS143-*araC-*P*_bad_* / pEVS143-*araC-*P*_bad_*-*opaR*-*5’UTR*-*gfp* | |
| FJS-S377 | | aagagacctagaactGGTACCATGTCTAAAGGTGAAG | Forward primer for insertion of the *gfpmut3* gene into pEVS143-*araC-*P*_bad_* / pEVS143-*araC-*P*_bad_*-*opaR*-*5’UTR*-*gfp* | |
| FJS-S378 | | tgctcaatcaatcaccggatcctggttgcgTTATTTGTATAGTTCATCCATGCC | Reverse primer for insertion of the *gfpmut3* gene into pEVS143-*araC-*P*_bad_* / pEVS143-*araC-*P*_bad_*-*opaR*-*5’UTR*-*gfp* | |
| FJS-q007 | | CCAAATGACAGCAGCGATAAG | Forward primer against the RIMD *ompW* gene | |
| FJS-q008 | | GAACATGTAGCCAAACGTCAAA | Reverse primer against the RIMD *ompW* gene | |
| FJS-q009 | | CTGCTGACTCTGATTGTGCTG | Forward primer against the φVP882 *gp69* gene | |
| FJS-q010 | | TCGTGAGAGGTGATGTACTTCTC | Reverse primer against the φVP882 *gp69* gene | |
| FJS-q001 | | TATGCGTGGCGGGTGAAA | Forward primer against the φVP882 *cos* site | |
| FJS-q002 | | AGCCCAAACCGACGGAAA | Reverse primer against the φVP882 *cos* site | |
| FJS-q053 | | CAACACCCAAGGCAATATACA | Forward primer against the φVP882 *IRS* site | |
| FJS-q054 | | CACACCTAGCCCTATACCTATG | Reverse primer against the φVP882 *IRS* site | |

*FJS-O primers were used for cloning. FJS-q primers were used for quantitative PCR.

**All sequences are listed in the 5’ to 3’ direction. Lowercase letters represent overlaps for homologous recombination. Underlined letters represent locations of point mutations. Bold letters represent synthetic ribosome binding sites.

**REFERENCES**

1. Hustmyer CM, Simpson CA, Olney SG, Rusch DB, Bochman ML, Van Kessel JC. Promoter Boundaries for the *luxCDABE* and *betIBA-proXWV* Operons in *Vibrio harveyi* Defined by the Method Rapid Arbitrary PCR Insertion Libraries (RAIL). J Bacteriol. 2018;200(11):e00724-17.
