# Supplementary material for: The LuxO-OpaR quorum-sensing cascade differentially controls Vibriophage VP882 lysis-lysogeny decision making in liquid and on surfaces
Source: PLoS Genet. 2024 Jul 30;20(7):e1011243. doi: 10.1371/journal.pgen.1011243 (PMC11315295; doi:10.1371/journal.pgen.1011243)
Supplement: S3 Table — (DOCX) [file pgen.1011243.s003.docx]

**S3 Table. Plasmids used in this study.**

| **Plasmid** | **Description** | **Identifier** | **Reference** |
| --- | --- | --- | --- |
| pRE112 | Sucrose counterselectable vector for allelic exchange ; Cm^r^ |  | (1) |
| pRE112-Δ*cpsA* | Allelic exchange vector for the clean deletion of *cpsA* (*VPA1403*) ; Cm^r^ | FJS-P025 | This study |
| pRE112-Δ*pomA* | Allelic exchange vector for the clean deletion of *pomA* (*VP0689*) ; Cm^r^ | FJS-P008 | This study |
| pRE112-*luxO^D61E^* | Allelic exchange vector for the introduction of the *luxO^D61E^* point mutation (*VP2099*) ; Cm^r^ | FJS-P013 | This study |
| pRE112-*luxO^D61A^* | Allelic exchange vector for the introduction of the *luxO^D61A^* point mutation (*VP2099*) ; Cm^r^ | FJS-P014 | This study |
| pRE112-Δ*opaR* | Allelic exchange vector for the clean deletion of *opaR* (*VP2516*) ; Cm^r^ | FJS-P015 | This study |
| pRE112-Δ*scrABC* | Allelic exchange vector for the clean deletion of *scrABC* (*VPA1513-VPA1511*) ; Cm^r^ | FJS-P037 | This study |
| φVP882::Cm^r^ | φVP882 with Tn5 inserted at a neutral locus. Tn5 includes Cm^r^ resistance cassette and an *oriT* site for conjugative transfer of the φVP882 genome | JSP-002 | (2) |
| pEVS143-*araC-*P*_bad_-vqmAφ* | Arabinose-inducible *vqmAφ* overexpression vector (P*_bad_*-*vqmAφ*) ; Kan^r^ | pJES-052 | (2) |
| pEVS143-*araC-*P*_bad_*-*q* | Arabinose-inducible *q* overexpression vector (P*_bad_*-*q*) ; Kan^r^ | pJES-093 | (2) |
| pXB300 | Tetracycline-inducible overexpression vector pXB300 ; Amp^r^ |  | (3) |
| pXBCm | Cm-resistant derivative of tetracycline-inducible overexpression vector pXB300 ; Cm^r^ | FJS-P061 | This study |
| pXBCm-*qrr*2 | Tetracycline-inducible *qrr*2 overexpression vector (P*_tet_*-*qrr*2) ; Cm^r^ | FJS-P063 | This study |
| pXBCm-*opaR* | Tetracycline-inducible *opaR* overexpression vector (P*_tet_*-*opaR*) ; Cm^r^ | FJS-P067 | This study |
| pXBCm-*scrABC* | Tetracycline-inducible *scrABC* overexpression vector (P*_tet_*-*scrABC*) ; Cm^r^ | FJS-P068 | This study |
| pXBCm-*scrC^E554A^* | Tetracycline-inducible *scrC^E554A^* (phosphodiesterase-null version of ScrC) overexpression vector (P*_tet_*-*scrC^E554A^*) ; Cm^r^ | FJS-P074 | This study |
| pXBCm-*tpdA* | Tetracycline-inducible *tpdA* overexpression vector (P*_tet_*-*tpdA*) ; Cm^r^ | FJS-P080 | This study |
| pXBCm-*gefA* | Tetracycline-inducible *gefA* overexpression vector (P*_tet_*-*gefA*) ; Cm^r^ | FJS-P081 | This study |
| pEVS143-P*_luxC_*-*luxCDABE* | Transcriptional reporter of the QS-regulated *luxC* promoter from *V. campbellii* strain BB120 fused to the *lux* operon ; Kan^r^ | FJS-P073 | This study |
| pEVS143-P*_qrr_*_1_-*luxCDABE* | Transcriptional reporter of the RIMD *qrr*1 sRNA promoter fused to the *lux* operon ; Kan^r^ | FJS-P075 | This study |
| pEVS143-P*_qrr_*_2_-*luxCDABE* | Transcriptional reporter of the RIMD *qrr*2 sRNA promoter fused to the *lux* operon ; Kan^r^ | FJS-P076 | This study |
| pEVS143-P*_qrr_*_3_-*luxCDABE* | Transcriptional reporter of the RIMD *qrr*3 sRNA promoter fused to the *lux* operon ; Kan^r^ | FJS-P077 | This study |
| pEVS143-P*_qrr_*_4_-*luxCDABE* | Transcriptional reporter of the RIMD *qrr*4 sRNA promoter fused to the *lux* operon ; Kan^r^ | FJS-P078 | This study |
| pEVS143-P*_qrr_*_5_-*luxCDABE* | Transcriptional reporter of the RIMD *qrr*5 sRNA promoter fused to the *lux* operon ; Kan^r^ | FJS-P079 | This study |
| pEVS143-P*_cpsA_*-*luxCDABE* | Transcriptional reporter of the RIMD *cpsA* promoter fused to the *lux* operon ; Kan^r^ | FJS-P072 | This study |
| pEVS143-P*_scrA_*-*luxCDABE* | Transcriptional reporter of the RIMD *scrA* promoter fused to the *lux* operon ; Kan^r^ | FJS-P059 | This study |
| pEVS143-P*_lafA_*-*luxCDABE* | Transcriptional reporter of the RIMD *lafA* promoter fused to the *lux* operon ; Kan^r^ | FJS-P018 | This study |
| pEVS143-*araC-*P*_bad_*-*opaR*-*5’UTR*-*gfp* | Translational reporter consisting of the *opaR* 5’ UTR fused to the *gfp* gene ; Kan^r^ | FJS-P082 | This study |
| c-di-GMP biosensor | pMMB67EH vector containing *turboRFP* under the control of three c-di-GMP riboswitches (Bc3-Bc5), constitutively expressed AmCyan for normalization, and the *hok/sok* region from pXB300 ; Gm^r^ | pFY4535 | (4) |

**REFERENCES**

1. Edwards RA, Keller LH, Schifferli DM. Improved allelic exchange vectors and their use to analyze 987P fimbria gene expression. Gene. 1998;207(2):149–57.
2. Silpe JE, Bassler BL. A Host-Produced Quorum-Sensing Autoinducer Controls a Phage Lysis-Lysogeny Decision. Cell. 2019;176(1–2):268-280.e13.
3. Bina XR, Wong EA, Bina TF, Bina JE. Construction of a tetracycline inducible expression vector and characterization of its use in *Vibrio cholerae*. Plasmid. 2014;76:87–94.

4. Zamorano-Sánchez D, Xian W, Lee CK, Salinas M, Thongsomboon W, Cegelski L, et al. Functional Specialization in *Vibrio cholerae* Diguanylate Cyclases: Distinct Modes of Motility Suppression and c-di-GMP Production. mBio. 2019;10(2):e00670-19.
