# Supplementary figures and images for: The LuxO-OpaR quorum-sensing cascade differentially controls Vibriophage VP882 lysis-lysogeny decision making in liquid and on surfaces
Source: PLoS Genet. 2024 Jul 30;20(7):e1011243. doi: 10.1371/journal.pgen.1011243 (PMC11315295; doi:10.1371/journal.pgen.1011243)

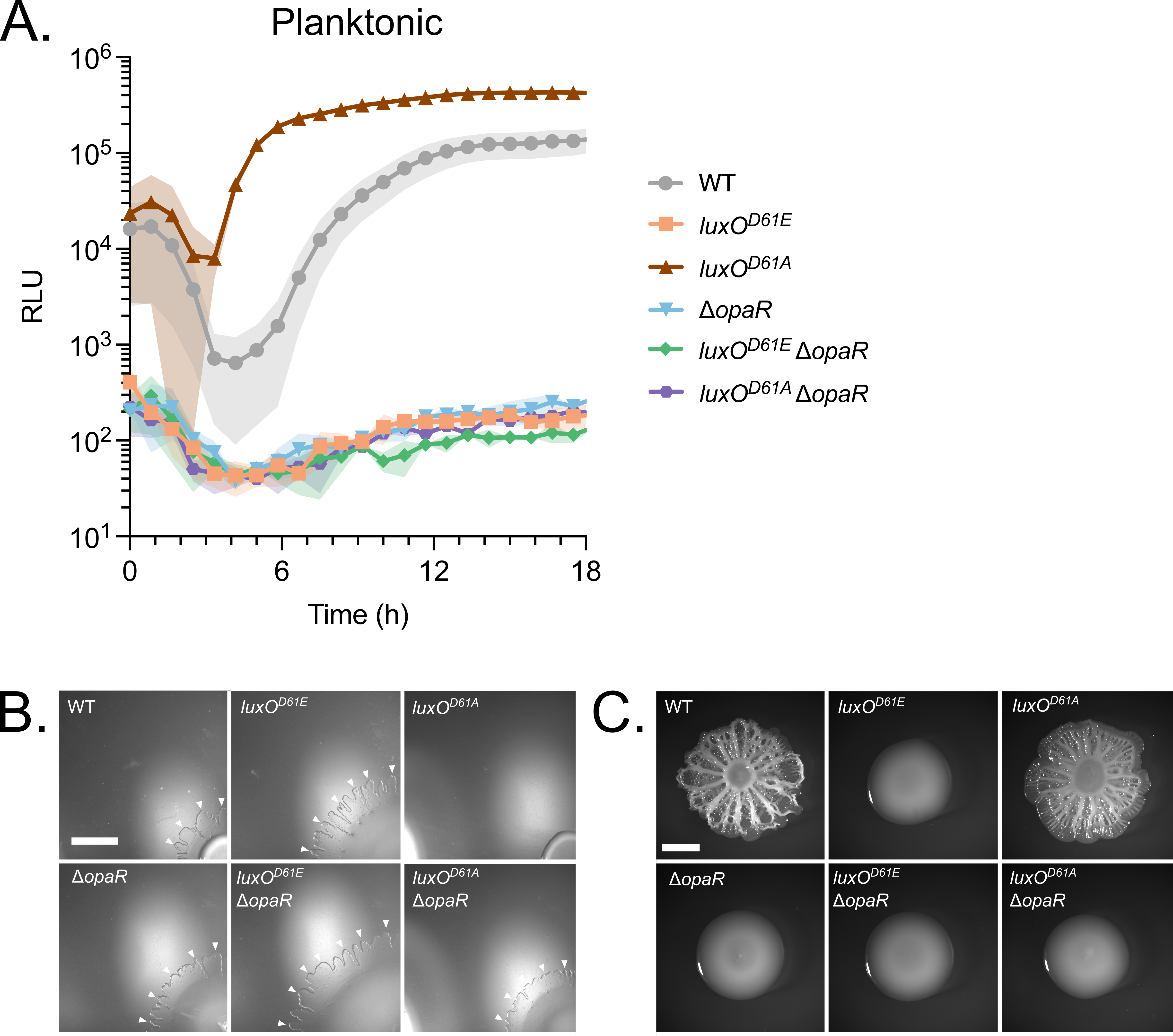

Supplement: S1 Fig — (A) Assessment of QS phenotypes in the indicated strains carrying a QS-activated lux reporter (PluxC-luxCDABE). Relative Light Units (RLU) are bioluminescence normalized to OD600. All experiments were performed in biological triplicate (n = 3). Lines and symbols represent the means and shaded areas represent the standard deviations. (B) Representative stereoscope images of swarming morphologies of the indicated strains 12 h post-inoculation. The bottom right corner of each image depicts the center of the colony. White arrows indicate the outer edges of the swarm flares in the WT and low-cell-density-locked strains. Swarming radius is measured from the bottom right corner of each image to the edge of the swarm flare. (C) Representative stereoscope images of biofilm morphologies for the indicated strains 42 h post-inoculation. (B,C) Scale bars = 3 mm. Swarming and biofilm phenotypes for the strains mirror those reported in the literature [10]. (TIFF) [file pgen.1011243.s005.tiff]

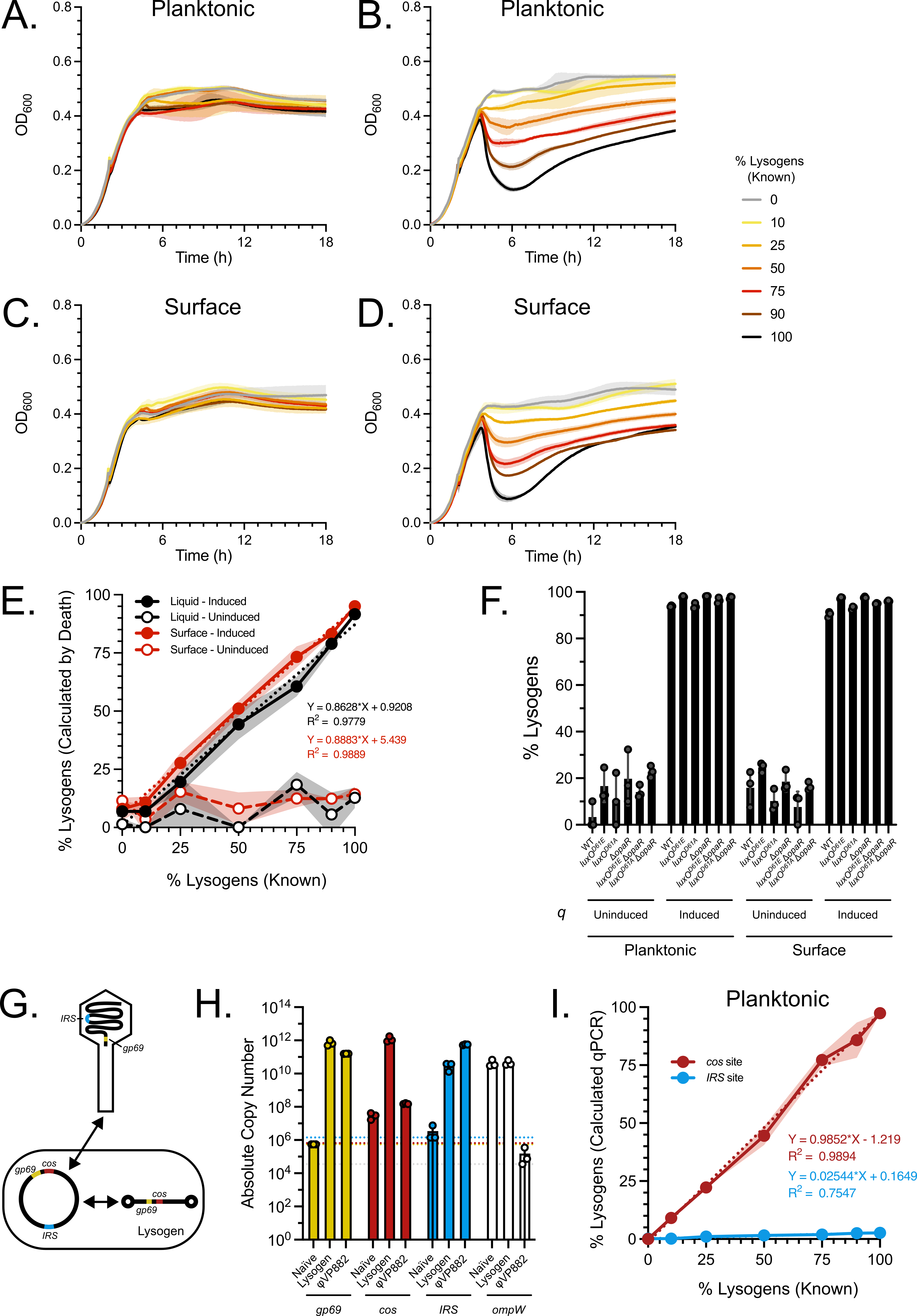

Supplement: S2 Fig — (A-D) Growth curves for WT RIMD populations consisting of known quantities of φVP882 lysogens and naïve host cells either (A,C) without q-induction (+dextrose) or (B,D) with q-induction (+arabinose). Strains were grown planktonically (A,B) or on a surface (C,D) prior to harvesting for the q-induction assay. Optical densities of samples in panels B and D at the peaks and valleys of their respective growth curves were used to calculate the lysogenized portion of the population (see Materials and Methods for a detailed explanation of the calculation). (E) Standard curves of the experimentally calculated percent lysogens versus the known percent lysogens without (+dextrose, open symbols) and with (+arabinose, closed symbols) q-induction for planktonically- (black) or surface-grown (red) cells. The dotted diagonal lines represent simple linear regressions performed on the induced samples. The resulting equations and R-squared values are shown. (F) Calculated percent lysogens in fully lysogenized populations of the indicated strains without (+dextrose) and with (+arabinose) q-induction after planktonic (left) or surface-associated (right) growth. (G) Diagram of the three genomic configurations of φVP882: the packaged linear form (top), the circular replicative form (bottom left), and the linear prophage form (bottom right). Shown are the gp69 gene (yellow), the cos site (red), and the IRS (blue). Double-sided arrows represent the flow of genomic rearrangements. (H) Absolute quantitation of the indicated φVP882 genomic regions in naïve cells, lysogens, and purified φVP882 particles. ompW is a gene in the RIMD genome used to calculate host genome copy number. Dotted lines represent the limit of detection for the DNA region with the corresponding color. (I) Standard curve of percent lysogens calculated by qPCR using primers against the φVP882 cos site (red) versus the known percent lysogens. Primers targeting the φVP882 IRS (blue) were included to demonstrate that the circul [file pgen.1011243.s006.tiff]

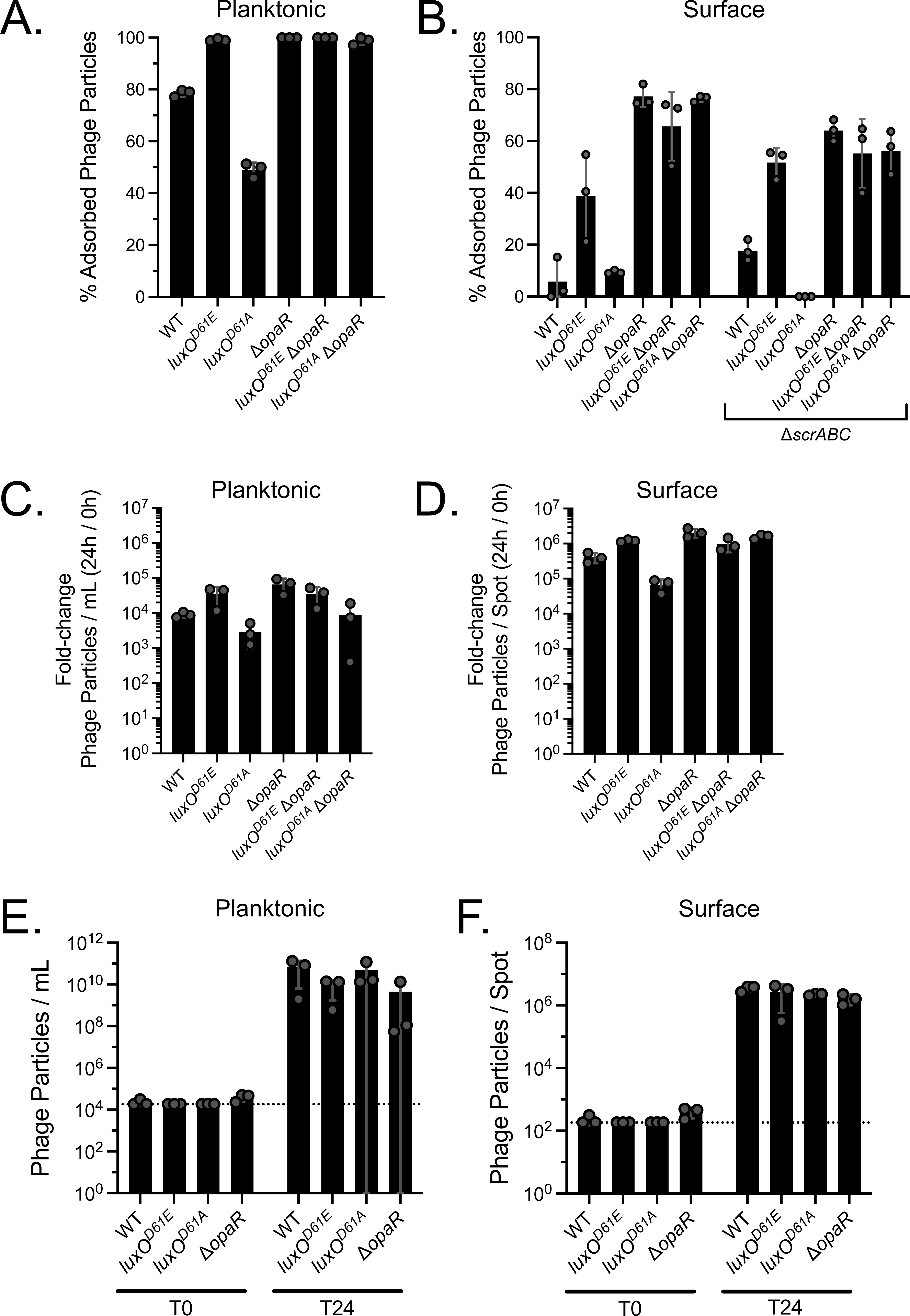

Supplement: S3 Fig — (A,B) φVP882 adsorption to the indicated RIMD strains shown as the percentage of phage particles removed by the cells from the culture medium after growth (A) in liquid or (B) on a surface. In A,B data are shown as 100%-% recovered phage particles. (C,D) φVP882 viral particle production in the indicated strains after infection (C) in liquid or (D) on a surface. Data are shown as the fold-change in harvested free viral particles at the end (24 h) of the infection compared to that at the beginning (0 h). (E,F) Quantitation of phage particles produced spontaneously from the indicated lysogenic strains when grown (E) in liquid or (F) on a surface. Dotted lines indicate the limit of detection by qPCR. (A-F) All experiments were performed in biological triplicate (n = 3). Symbols represent individual replicate values. Bars represent means. Error bars represent standard deviations. (TIFF) [file pgen.1011243.s007.tiff]

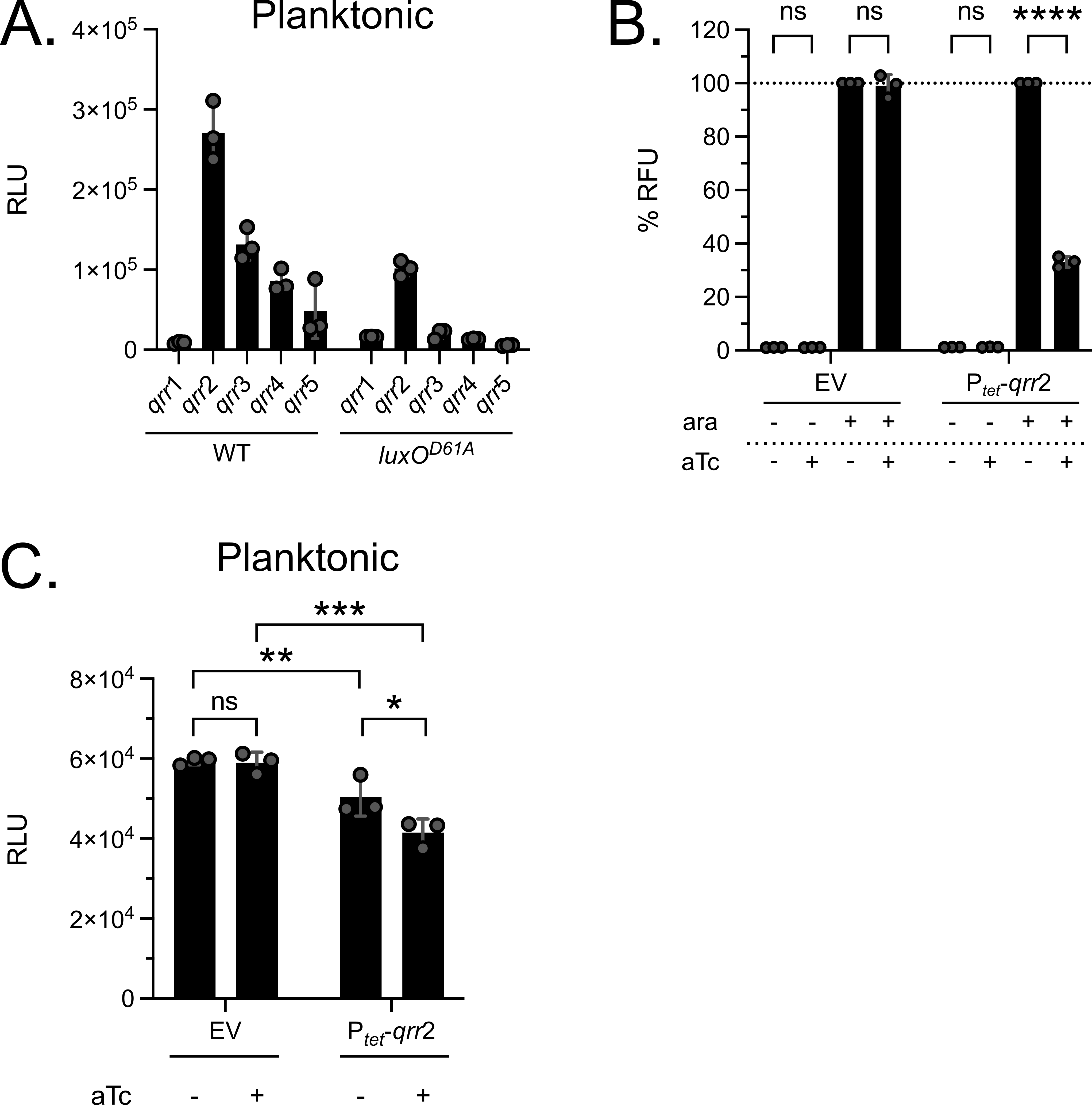

Supplement: S4 Fig — (A) Light production from transcriptional reporters of the five Qrr sRNA promoters (Pqrr1-5-luxCDABE) in WT RIMD and the high-cell-density-locked luxOD61A strain during planktonic growth. (B) Fluorescence output is shown from E. coli strains carrying an opaR-5’UTR-gfp translational reporter (Pbad-opaR-5’UTR-gfp) and either an empty vector control (EV) or a qrr2 overexpression construct (Ptet-qrr2). The opaR-5’UTR-gfp translational reporter was uninduced (+dextrose) or induced (+arabinose) in the absence (-aTc) or presence (+aTc) of qrr2 overexpression. Data (% RFU) are represented as percent GFP signal for each sample compared to the sample following induction of only the opaR-5’UTR-gfp translational reporter. (C) Light production from a transcriptional reporter of a QS-activated promoter (PluxC-luxCDABE) in RIMD carrying either an empty vector control (EV) or a qrr2 overexpression construct (Ptet-qrr2) is shown. Samples were uninduced (-aTc) or induced (+aTc). (A-C) All experiments were performed in biological triplicate (n = 3). Symbols represent individual replicate values. Bars represent means. Error bars represent standard deviations. (A,C) RLU are bioluminescence normalized to OD600. (B,C) Significance was determined by two-way ANOVA with Tukey’s multiple comparisons test to determine adjusted p-values: (B) ns = non-significant, **** p <0.0001, (C) ns = non-significant, * p = 0.0101, ** p = 0.0098, *** p = 0.0002. (TIFF) [file pgen.1011243.s008.tiff]

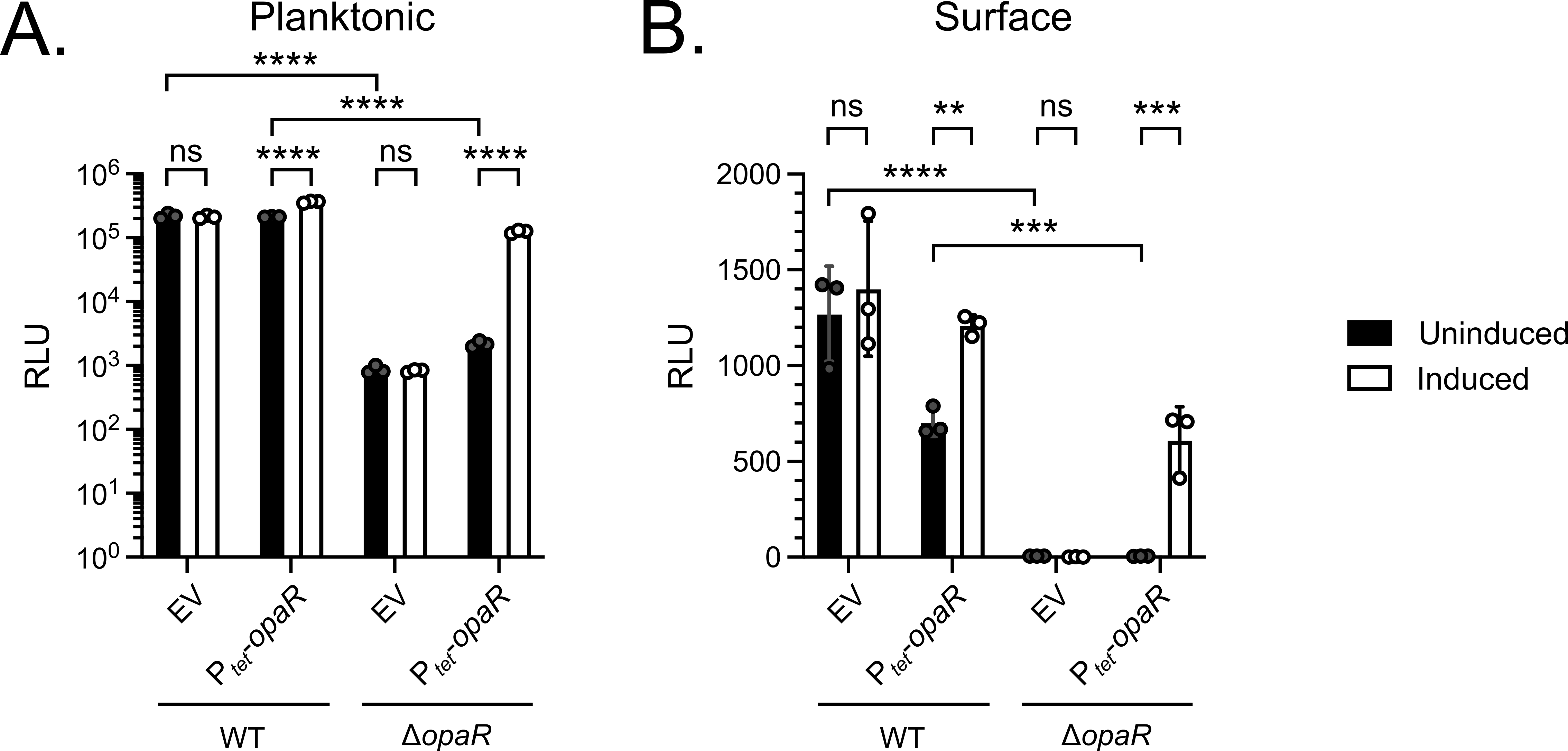

Supplement: S5 Fig — (A) Light production from a transcriptional reporter of the QS-activated luciferase operon (PluxC-luxCDABE) in planktonic RIMD strains carrying either an empty vector control (EV) or an opaR overexpression construct (Ptet-opaR). RLU are bioluminescence normalized to OD600. (B) Light production from a transcriptional reporter of the QS-activated exopolysaccharide operon (PcpsA-luxCDABE) in surface-associated RIMD strains carrying either EV or Ptet-opaR. RLU are bioluminescence normalized to constitutive mScarlet-I signal. (A,B) All experiments were performed in biological triplicate (n = 3). Symbols represent individual replicate values. Bars represent means. Error bars represent standard deviations. Samples were uninduced (-aTc, black) or induced (+aTc, white). Significance was determined by two-way ANOVA with Tukey’s multiple comparisons test to determine adjusted p-values: (A) ns = non-significant, **** p <0.0001, (B) ns = non-significant, ** p = 0.0019, *** p = 0.0005, 0.0004, **** p <0.0001. (TIFF) [file pgen.1011243.s009.tiff]

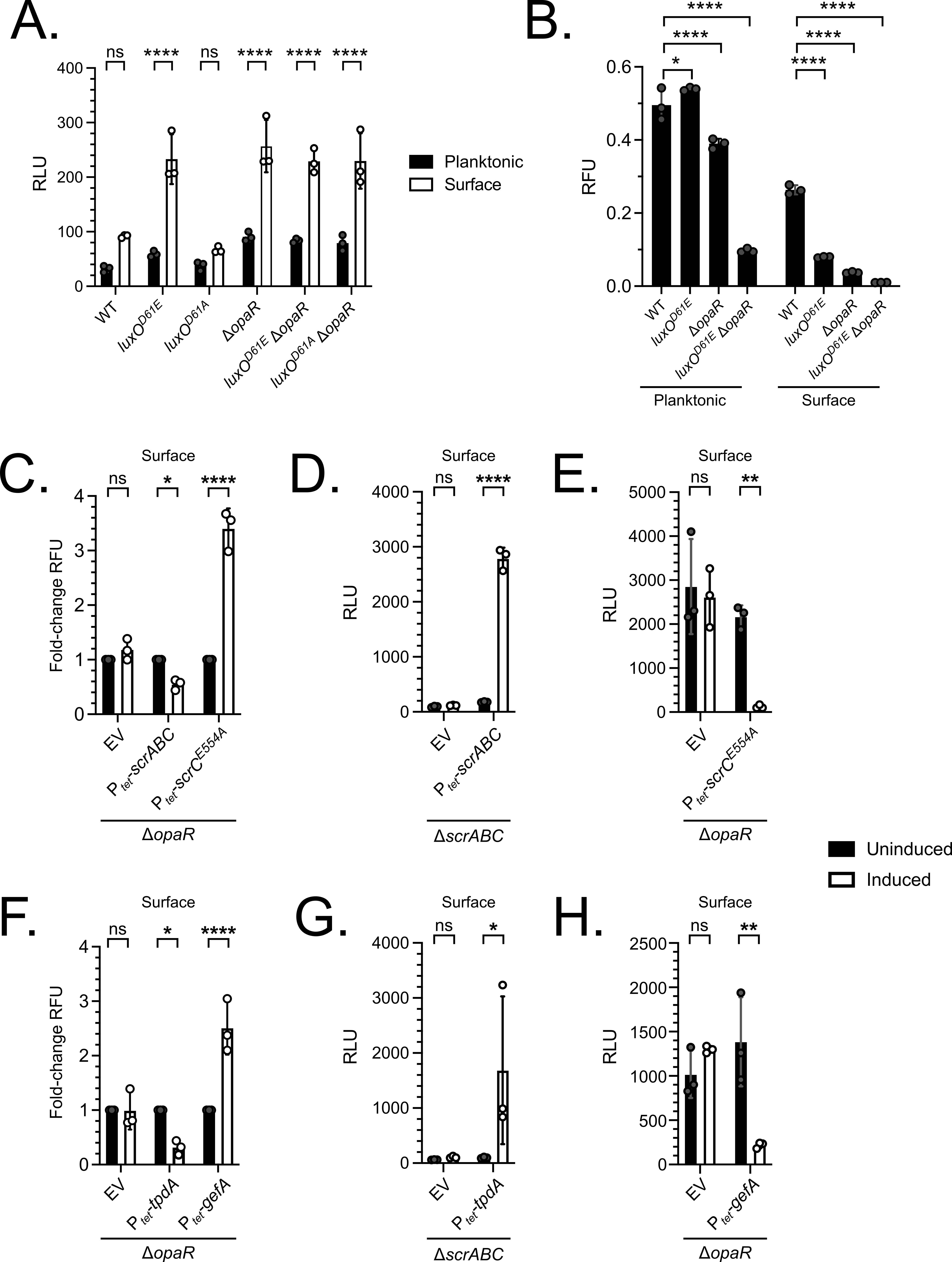

Supplement: S6 Fig — (A) Light production from a transcriptional reporter of the scrABC surface-sensing operon (PscrA-luxCDABE) in planktonic (black) and surface-associated (white) RIMD strains. (B) Relative c-di-GMP abundance across the indicated strains grown either in liquid or on a solid surface. (C) Fold-change in c-di-GMP abundance in surface-associated ΔopaR RIMD carrying either an empty vector control (EV), an inducible scrABC operon (Ptet-scrABC) in which ScrC functions as a phosphodiesterase, or an inducible allele of scrC (Ptet-scrCE554A) in which ScrC functions as a diguanylate cyclase. (D) Light production from a transcriptional reporter of the lateral flagellin promoter (PlafA-luxCDABE) in surface-associated ΔscrABC RIMD carrying either EV or Ptet-scrABC. (E) Light production from PlafA-luxCDABE in surface-associated ΔopaR RIMD carrying either EV or Ptet-scrCE554A. (F) Fold-change in c-di-GMP abundance in surface-associated ΔopaR RIMD carrying either EV, an inducible Ptet-tpdA construct encoding the TpdA phosphodiesterase, or an inducible Ptet-gefA construct encoding the GefA diguanylate cyclase. (G) Light production from PlafA-luxCDABE in surface-associated ΔscrABC RIMD carrying either EV or Ptet-tpdA. (H) Light production from PlafA-luxCDABE in surface-associated ΔopaR RIMD carrying either EV or Ptet-gefA. (A-H) All experiments were performed in biological triplicate (n = 3). Symbols represent individual replicate values. Bars represent means. Error bars represent standard deviations. (C-H) Black and white bars are uninduced (-aTc) and induced (+aTc), respectively. (A,D,E,G,H) RLU are bioluminescence normalized to constitutive mScarlet-I signal. (B,C,F) RFU is c-di-GMP controlled TurboRFP fluorescence normalized to constitutive AmCyan fluorescence. (C,F) aTc-induced values (white) are represented as the fold-change versus their corresponding uninduced values (black). (A,C-H) Significance was determined by two-way ANOVA with Sidak’s multiple comparisons test to determine [file pgen.1011243.s010.tiff]
