# Supplementary material for: The LuxO-OpaR quorum-sensing cascade differentially controls Vibriophage VP882 lysis-lysogeny decision making in liquid and on surfaces
Source: PLoS Genet. 2024 Jul 30;20(7):e1011243. doi: 10.1371/journal.pgen.1011243 (PMC11315295; doi:10.1371/journal.pgen.1011243)
Supplement: Attachment — Submitted filename: Santoriello2024_ResponseToReferees.pdf [file pgen.1011243.s011.pdf]

As you will see below, all three referees were enthusiastic about this submission and believe it is a highly valuable contribution to the field. But they also highlight a number of issues that need to be addressed. In particular, the reviews highlight concerns with the newly developed Q-induction assay used to assess lysogeny, and have provided actionable suggestions on how to address these concerns and further validate the use of this assay with another approach.

We thank the referees for their enthusiasm and especially for their thoughtful recommendations for improvement. We have taken the referee comments to heart and changed the manuscript exactly as directed. We performed several new experiments and included the additional data, we strengthened data in other sections, we clarified our writing in several places, and we validated our methods. We hope that you and the referees now find the manuscript suitable for publication.

Comments to the Authors:
Please note here if the review is uploaded as an attachment.

Referee #1: Major comments
1. Despite the argument presented for why the large differences in phage adsorption efficiency between strains don't impact the results, at a minimum, it clouds interpretation of the data. Being only a 10-minute phage adsorption assay (Figure S3), one must suspect significant differences in phage receptor expression between strains. However, nowhere in the manuscript is the receptor even mentioned. What is the receptor, and does its expression vary between the different strains tested? Is receptor expression cell density dependent, or planktonic versus surface dependent? Why wasn't phage adsorption measured for surface-grown strains?

We thank the referee for pointing out that this portion of our results lacked clarity. The φVP882 receptor is not known so we could not discuss it nor investigate its production pattern. The data arguing against adsorption differences among strains driving the observed differences in lysogenic conversion of planktonic cells lies in comparisons of the *luxO*^{D61E}, ∆*opaR*, *luxO*^{D61E} ∆*opaR*, and *luxO*^{D61A} ∆*opaR* mutants. Over 98% adsorption occurs to each of these strains, and yet, lysogens are only detected in strains carrying the *luxO*^{D61E} mutation. Thus, a mechanism other than adsorption must dictate the propensity for lysogenic conversion. We lay out this reasoning in the text and show the results in Fig. S3A.

To address surface adsorption, we performed the adsorption assay with cells grown on filters on agar surfaces. Our new data (Fig. S3B) show that, analogous to the planktonic cell adsorption results (Fig. S3A), φVP882 can adsorb to each strain, albeit with less affinity to the WT and *luxO*^{D61A} mutants than to the strains locked at low cell density. Importantly, deletion of the *scrABC* operon does not affect the ability of φVP882 to adsorb, indicating that the observed loss of lysogenic conversion in surface-associated cells lacking *scrABC* is not due to reduced viral attachment.

2. The Discussion section states several times that the phage can adsorb to cells grown at any density, but how can this be, given that it does not absorb well to WT and luxOD61E in Figure S3? Perhaps you mean that it can adsorb to at least a fraction of the cells regardless of culture density.

Based on this comment, we have clarified the text regarding the adsorption results. We have modified Fig. S3A, so it now shows the percentages of adsorbed phages for each strain rather than the quantity of remaining phages. Depicting the data in this way makes it readily evident that, within 10 min, 80% and 50% of the added phage particles have adsorbed to the WT and $luxO^{D61A}$ strains, respectively.

3. The Q-induction assay for lysogeny of surface-grown bacteria needs to be validated in some way, as was done for planktonic cells. Otherwise, how can we trust the lysogeny data presented?

As suggested, we performed *q*-induction validation experiments with surface-grown strains. Surface-grown cells produced a standard curve of Q-directed cell death with a slope nearly identical to that obtained for planktonic cells (standard curves m = 0.89 and m = 0.86, respectively; Fig. S2C-E). We also verified that none of the QS mutations affect *q*-induction in cells harvested from surfaces (Fig. S2F). Thus, the *q*-induction assay is accurate for both planktonic and surface-associated cells.

Minor comments
Lines 125, 211, 218, etc: Your use of “surprisingly” is not scientific and distracts the reader. I would delete all such uses.

All instances of “surprisingly” have been removed.

Line 171: This is more likely due to a polar effect of the antibiotic resistance cassette. Either way, such speculation isn’t necessary. Replace with “due to unknown reasons.”

The text has been modified as follows:

“Regarding lysogenic conversion, for reasons we do not understand, insertion of an antibiotic resistance cassette into the φVP882 genome resulted in a strong defect in phage infectivity.”

Line 367: Missing a hyphen.

The typo has been fixed. Thanks, good eye.

Referee #2: The authors present a thorough and deep assessment of the role of quorum sensing in VP882 phage lysis-lysogeny decision. Through very detailed and thorough genetic experiments, the authors demonstrate two key findings: 1) the QS circuit components OpaR and the Qrrs regulate lysogeny at HCD and LCD, respectively, thus only at LCD will lysogeny occur, and 2) the surface-sensing system of ScrABC blocks lysogeny on surfaces at HCD, also controlled by OpaR. I am impressed with the fine-level detail of the genetic dissection of the pathways, as well as the assays that validate the QS states, measurements of lysogeny, expression of Qrr2, and measurements of cdGMP levels. I have only a few comments for clarity and an experimental suggestion.

Major comments:

• Line 173: I wondered when reading: why did the authors not measure lysogeny vs lysis by qPCR using the formation/level of attL or attR vs attP. I understand that the authors developed the Pbad-q assay to measure lysis to calculate lysogenic conversion, and that works here. But it may be useful (either in this manuscript or in the future) to use the above-mentioned method for direct comparison of lysis and lysogeny both by qPCR (which can then also measure copy number during lytic replication).

Thank you for suggesting qPCR as a method to assess lysogenic conversion. φVP882 is a linear plasmid phage that is maintained as a multicopy, extrachromosomal, element. Thus, there is no *attL/R* or *attB/P* site. For that reason, we performed absolute quantitation against the φVP882 *cos* site, a sequence unique to the φVP882 prophage (Fig. S2G,H). All *cos* site counts were normalized to the *cos* site copy number per cell for a stable φVP882 lysogen grown under the same conditions [(total *cos* sites / *cos* site copy per cell) / total host cells]. A qPCR standard curve of lysogen/non-lysogen mixtures (Fig. S2I) delivered a slope nearly identical to that obtained when we used our *q*-induction method (Fig. S2E). Thus, the *q*-indication method accurately quantifies lysogenic conversion.

• Line 206: When infecting RIMD strains, what OD were they at in the beginning of the experiment? Methods say 1:100 dilution of an overnight culture. I suspect this isn't actually "LCD" because the WT behaved like the HCD strain, and thus that result implies that the density of the infected cultures was similar to the HCD state. This should be clarified in the results. Would the result be different if the overnight cultures were diluted 1:10,000? (Line 521 methods). This seems likely as this is what appears to happen in subsequent experiments (lines 252, 282).

This comment and that from referee 3 inspired us to examine MOI in our infection experiments. We initially quantified our phage stocks by qPCR yielding ~3e11 particles/mL. However, by plaque assay, we calculate ~2e6 PFU/mL. This discrepancy stems from differences in total particles and in infective particles in our stocks. The finding means that in the original version of the manuscript, our infection experiments were performed at an MOI ~ 10^{-5}. Thus, the WT cultures were able to reach high cell density before the phage could reach the critical MOI required for lysogenic conversion. In the revised version of the manuscript, our examination of MOIs ranging from 10^{-2} to 10^{-5} and performing infections for 24 h (Fig. 2B) revealed that strains carrying WT *luxO* show MOI-dependent lysogenic conversion, while strains carrying the low-cell-denisty-locked $luxO^{D61E}$ allele exhibit MOI-independent lysogenic conversion. This result suggests that all strains are at low cell density at the start of our experiments. Moreover, for φVP882-directed lysogenic conversion of RIMD to occur, a sufficiently high MOI must exist concurrent with the low-cell-density QS status of the cells (Fig. 2B,C). We go on to show that, when the MOI condition is met, it is the presence of high level Qrr sRNAs that drive lysogenic conversion at low cell density (Fig. 2D,E).

We are grateful to the referees for giving us this new idea. The additional experiments deepened the work and allowed us to expand our understanding of the mechanisms that interact to drive the lysogenic conversion process.

• Line 333: This should be a new results subsection connecting all the cdGMP results.

Thank you for this suggestion. A new results section was added with the following title:

"Degradation of the global c-di-GMP pool leads to increased φVP882 lysogenic conversion of RIMD"

Minor comments:
• Lines 263-269 seem repetitive with the info in the introduction.

We shortened this section while keeping some of the original information. We hope the referee can agree that it is helpful for readers less familiar with the topic to have this bit of information to transition from the planktonic cell data to the surface-grown cell data.

• It would help with reading/clarity if the graphs throughout were labeled with "planktonic" or "surface-associated".

Agreed. As the referee suggests, we have labeled all graphs with either "planktonic" or "surface".

Referee #3: Review of the manuscript “The LuxO-OpaR quorum-sensing cascade differentially controls Vibriophage VP882 lysis-lysogeny decision making in liquid and on surfaces” by Santoriello and Bassler

Summary of the manuscript

This work follows on previous works of the Bassler lab which have shown that phage phiVP882 can switch from lysogeny to lysis through sensing of a quorum-sensing signal (DPO) produced by the host. This occurs through activation of a phage encoded receptor (phiVqmR) homologous to the host receptor which senses the signal and leads to the production of an antirepressor. In a follow-up work they have also shown that this induction is enhanced by a factor regulated by other QS systems of the host (LuxO).

In this work, Santoriello and Bassler set out to find host factors which affect the lysis-lysogeny decision of the phage upon infection. Lacking the ability to perform PFU and CFU measurements, they have developed alternative assays to assess the number of lytic phages and the frequency of lysogens in a population. They found that LuxO activity, regulated by the triple converging QS systems characterizing many vibrios (and studied by the Bassler lab), is a strong modulator of the level of lysogeny, with lysogeny enhanced by conditions of low cell density. They find that this effect is differentially regulated in liquid medium and in surface association. In liquid medium, this effect requires only LuxO~P dependent activation of the small qrr RNAs, while in surface association LuxO~P transduces the signal to OpaR which controls c-di-GMP levels through the Scr system, and lysogeny is induced at low c-di-GMP level.
General assessment

This is a very intriguing work, and is definitely worth publishing in PLOS Genetics, once all experimental issues are resolved. However, there are several major issues with the lysogeny assay used by the authors which will require further validation of the results. There are also several issues with the interpretation of the results and their presentation.

Major issues - Lysogeny assay
==================

To assess lysogenization rate (or propensity), the authors perform the following assay (as detailed in the methods section). They dilute ON grown bacteria 1:100 and infect them at a MOI of 0.1. They then let bacteria and phages grow for 24 hours.
All mutations where done in a strain encoding the pro-lytic q gene on an IPTG inducible promoter. After the 24 hours of growth, cells are diluted 1:100 into media where phages cannot infect and grown further to mid-log, prior to induction of the host-encoded q gene by IPTG and assessment of lysogeny.
There are several issues with this assay as I discuss below:

1. Does this assay actually measure lysogenization frequency at all? Phages and bacteria are left for 24 hours after infection, during this time there is clearly a lot of

infections occurring resulting in lysogeny, but also in a lot of lysis and re-infection. The assay therefore is a cumulative measurement of lysogeny during the whole infection process. The final number (and maybe also frequency) of lysogens in such an assay is optimized by mutants which are not fully lysogenic. In fact, a mutant which leads to full lysogeny and infected at MOI of 0.1 would necessarily result in 10% frequency of lysogens and not in 100%, as all infections would immediately lead to lysogeny. Therefore, in this case, the strain with lower lysogenization frequency would achieve a higher score, showing that this assay may be flawed in principle.
It is important to try and assay the impact of the mutants on lysogeny in a single round of infection. I suggest allowing the cells and phages to interact for a limited time (say 30 minutes) and not for 24 hours to assess the level of lysogenization there. Now, I am aware of the fact that the frequency of resulting lysogens may be too low to appropriately measure with the q induction method, but it is worth trying. Additionaly, I suggest adding a different, complementary, assay in any case, as detailed below.

We thank the referee for bringing up this excellent point concerning possible mechanisms underlying our results.

In response to the referee's point regarding whether particular mutants are fully lysogenic and incapable of launching lytic induction after infection, in the revised work we quantified spontaneous phage production from lysogenized WT, *luxO*^{D61E}, Δ*opaR*, and *luxO*^{D61A} strains (Fig. S3E,F). Our results show that RIMD undergoes similar amounts of spontaneous induction at all cell densities, and thus, no strain is locked into a fully lysogenic state.

As the referee notes, the best way to assess the influence of QS on lysogeny is to measure the frequency of lysogenic conversion after a single round of infection. As suggested, we performed these analyses and we have included them in the revised manuscript. Specifically, we infected our set of RIMD strains and measured the frequency of *cos* sites by qPCR after one infection cycle (Fig. 2D). The results show that the low-cell-density-locked strains are lysogenized twice as frequently as are the high-cell-density-locked strains. Indeed, this pattern persists beyond one infection cycle (Fig. 2D). These new results support the data we obtained for lysogenic conversion using the *q*-induction assay.

2. Why are there non-lysogens at all? The measurement of lytic particles suggests that viral particles reach very high numbers by 24 hours of infection. If this is the case, it is not clear at all to me why non-infected cells remain, as we would expect only lysogens to be resistant to infection. I could see several options. First, the non-infected cells may be mutants that arose during the 24 hours. Second, the community might go into a non-infective state at a certain timepoint (e.g, upon entering stationary) maintaining cell that are uninfected, though sensitive. Further, in some mutants 100% of bacteria became lysogens, indicating that all cells have been infected. If there is no major difference in the ability of phages to infect the different mutants (as claimed in the manuscript), how is it that in certain mutants there remain uninfected cells but not in others? Specifically,

the mutants which most increase lysogenization frequency are those that show 100% infection, which adds to the mystery.
It is important to distinguish between the above options. With the current assay of 24 hours of infection, it may be that a major effect of the different host mutations is the time when the cells enter the non-infected state, or the tendency of resistance mutants to arise in different host background and not the tendency to lysogenize.
Also, in this respect, it would be good to provide some understanding of the total number of cells that survive after 24 hours of infection and not only the frequency of infected and non-infected cells. A supp figure of a plate reader graph showing infections of WT, high cell density, and low cell density mutants in liquid would also help understand the difference between the strains.
Again, all of these issues stress the importance of monitoring a single infection round and not the result of a culminative infection process after 24 hours.

We agree with the arguments the referee makes. Indeed, we have many of the same questions. Unfortunately, the lytic dynamics of linear plasmid prophages, e.g. φVP882, have not been well studied. Under our infection conditions, new phage particles are produced, but no detectable reduction in optical density occurs. We do not understand the biology underlying this phenomenon, and to our knowledge neither does anyone else. We have yet to find a report to explain it. We are grateful for the referee's suggestion to monitor early rounds of infection. As described in response to this referee's point 1, we did so. That idea enabled us to validate that the patterns of lysogenic conversion of the different RIMD strains that are measured by our *q*-induction assay (Fig. 2B,C) are established in the first round of infection (Fig. 2D).

3. How sensitive is the q-induction assay? The authors perform the appropriate calibration of the assay to a set of cocultures of lysogens and non-lysogens at different frequencies. However, given the limits of the system (low reliability below ~5-10%), I think it would be informative to compare these results (at least of several selected mutants, with a clear difference in lysogenization frequency) to alternative quantification methods. For example, the authors can perform a qPCR (of the same gene they use for assaying lytic particles) on the genomic extraction of a population. I would expect to see clear correlation between the frequency of lysogens in the two assays. The qPCR assay may be more reliable at low frequencies and may therefore allow measuring lysogeny after a single round of infection.

We thank the referee for raising this concern. We recognize that qPCR is more sensitive for detection of φVP882 lysogens than is our *q*-induction assay. One of our main motivations for developing the *q*-induction assay was to enable our investigations at the high throughput required to cope with the large numbers of mutants and conditions under study. qPCR is not possible for the hundreds of measurements made in this work. Nonetheless, we have included the qPCR validation of the *q*-induction method as it significantly bolsters the strength of those results.

Regarding the qPCR method: we performed absolute quantitation against the φVP882 *cos* site, a sequence unique to the φVP882 prophage (Fig. S2G,H). All *cos* site counts

were normalized to the *cos* site copy number per cell for a stable φVP882 lysogen grown under the same conditions as the strain under study and the number of host cells [(total *cos* sites / *cos* site copy per cell) / total host cells]. A qPCR standard curve of lysogen/non-lysogen mixtures (Fig. S2I) resulted in a similar slope to the *q*-induction standard curve (Fig. S2E). Importantly, the qPCR method allowed detection of lysogeny in the 0-10% range, making the single round of infection experiments possible (Fig. 2D).

Minor issues
========

1. Given the importance of the Vqm system to induction, it is surprising that the authors haven't checked the impact of the host Vqm system on lysis-lysogeny. Similarly, does addition of DPO affect the decision? This is not necessary for the manuscript, but would improve it.

The host VqmA-VqmR system has not been shown to play any role in φVP882 lysis-lysogeny decision making. These findings were reported previously:

- Duddy et al., 2021 (doi: 10.1371/journal.pgen.1009550) showed that host VqmA cannot activate expression of the *qtip* gene encoding the phage antirepressor.

- Duddy et al., 2023 (doi: 10.1371/journal.pgen.1010809) showed that the host VqmA-VqmR system had a minimal effect on lytic induction of φVP882.

2. It is also surprising that the wild-type is non-lysogenic. My guess is that a 100-fold dilution from overnight never allows the cells to really go into the LCD state? It might be interesting (but not necessary) to dilute much more (say 10,000) and let the cells grow for some time to reach the same density for infection and monitor lysogeny under these conditions.

This comment and that from referee 2 inspired us to examine MOI in our infection experiments. We initially quantified our phage stocks by qPCR yielding ~3e11 particles/mL. However, by plaque assay we calculate ~2e6 PFU/mL. This discrepancy stems from differences in total particles and in infective particles in our stocks. The finding means that in the original version of the manuscript, our infection experiments were performed at an MOI ~ 10^{-5}. Thus, the WT cultures were able to reach high cell density before the phage could reach the critical MOI required for lysogenic conversion. In the revised version of the manuscript, our examination of MOIs ranging from 10^{-2} to 10^{-5} and performing infections for 24 h (Fig. 2B) revealed that strains carrying WT *luxO* show MOI-dependent lysogenic conversion, while strains carrying the low-cell-denisty-locked *luxO*^{D61E} allele exhibit MOI-independent lysogenic conversion. This result suggests that all strains are at low cell density at the start of our experiments. Moreover, for φVP882-directed lysogenic conversion of RIMD to occur, a sufficiently high MOI must exist concurrent with the low-cell-density QS status of the cells (Fig. 2B,C). We go

on to show that, when the MOI condition is met, it is the presence of high level Qrr sRNAs that drive lysogenic conversion at low cell density (Fig. 2D,E).

We are grateful to the referees for giving us this new idea. The additional experiments deepened the work and allowed us to expand our understanding of the mechanisms that interact to drive the lysogenic conversion process.

3. Interpreting the data: The authors seem to suggest that the phage directly sense the genetic states of the cells through sRNA and c-di-GMP levels. However, other interpretation of the data may also be possible. Specifically, the phage may sense a more general physiological state that is associated with the different genetic states. For example, phage lambda is known to modulate its lysis/lysogeny decision based on the growth rate of the cell through the RnaseIII system. It may very well be that the specific genetic states (LCD, HCD, surface biofilm, surface swarming etc) lead to a difference in cellular physiology (e.g, growth rate) which is sensed by a single mechanism similar to the RNAseIII system. Distinguishing between the alternatives goes beyond the scope of the manuscript, but this should be mentioned in the discussion.

We thank the referee for pointing out this phenomenon. We agree there are alternative possibilities. We have added this point to the revised discussion:

"Alternatively, it is possible that the connection between LuxO phosphorylation/dephosphorylation and φVP882 lysis-lysogeny transitions is indirect and due to φVP882 detection of a shift in general host physiology that is triggered by both the Qrr sRNAs in planktonic cells and decreased c-di-GMP in surface-associated cells."

4. Figures
a. The "uninduced q" bars and "uninduced plasmid" bars only serve as controls for the leakiness of the q construct and the inducible plasmids. Therefore, their results should be moved to Supp. Figures. Currently, they just distract from the main results.
b. Fig. 4A is very confusing with the two colors for the two different induced plasmids and given that some of the bars are very near zero and it is hard to distinguish between the different colors. I would consider revising the figure.

Indeed, good idea as it makes the data easier to follow. We have removed all "+dextrose" data from the manuscript. These data are now only included in the Supporting Data file. Also, Fig. 4A,C have been modified for clarity.

5. Lines 412-415. I think that the authors want to say: "Taken together with our current findings, it appears that phosphorylation/dephosphorylation of LuxO enables toggling between φVP882 !lysogeny and lysogeny! (via Qrr sRNAs or ScrABC, depending on the physical context of the host) and !lytic induction! (via DPO-VqmAφ) (Fig. 5)"
Note: Changes are marked by ! signs on both sides.

Thank you for this suggestion. This is not quite the idea we were attempting to convey. The text has been modified as follows for clarity:

"While not investigated here, it is possible that LuxO-dependent regulation of lysogenic conversion and VqmAφ-dependent regulation of lytic induction are connected. Specifically, phosphorylation/dephosphorylation of LuxO may enable toggling between φVP882 lysogeny (via Qrr sRNAs or ScrABC, depending on the physical context of the host) and lysis (via DPO-VqmAφ) both when the initial lysis-lysogeny decision is made and later in infection when the decision to transition from lysogeny maintenance to lytic induction is undertaken (Fig. 5)."
